# Supplementary material for: Examining gender inequalities in factors associated with income poverty in Mexican rural households
Source: PLoS One. 2021 Nov 4;16(11):e0259187. doi: 10.1371/journal.pone.0259187 (PMC8568291; doi:10.1371/journal.pone.0259187)
Supplement: Attachment — Submitted filename: Response to Reviewers.docx [file pone.0259187.s007.docx]

Department of Economics

Georg-August-Universität Göttingen

Platz der Göttinger Sieben 3

37073 Göttingen

Germany

July 5, 2021

Dear Carlos Alberto Zúniga-González,

We would like to thank you very much for inviting us to revise and resubmit our manuscript “Examining gender inequalities in factors associated with income poverty in Mexican rural households”. We appreciate the time and effort that you and the reviewers have dedicated to providing insightful comments and suggestions that have helped us to improve the quality and readability of the manuscript.

Following your advice, we have clarified the main research questions and framed the contributions of the paper to the existent literature on poverty as well as modified the wording to make it less technical and more comprehensible to readers. In what follows, we provide detailed response to each comment, indicating the specific changes made in the manuscript to address them and the location where those are placed in the revised manuscript. In order to facilitate the follow-up of these changes, we have included line numbers in the revised manuscript with track changes.

We hope that the revised version fulfils your expectations and that the new structure and organization of the paper is now clearer.

Sincerely,

The authors

**Comments from the Academic Editor**

| **Comment 1:** | Please ensure that your manuscript meets PLOS ONE's style requirements, including those for file naming. |
| --- | --- |
| **Response:** | Thank you. We revised again the documents following the sample files for the title, author list, and affiliations page; and the manuscript body to ensure that our submission meets the formatting requirements of the journal.  As part of these changes, we shortened the title of the manuscript to “Examining gender inequalities in factors associated with income poverty in Mexican rural households” (before “Examining gender inequalities in factors associated with income poverty in Mexican rural households by a boosting additive quantile model with stability selection”) aiming to make it more specific, descriptive, concise, and comprehensible to readers outside the field, as required in the guidelines for the title, and to highlight that the main goal of the manuscript rests primarily in study of poverty and not in this specific analytical approach. |
|  |  |
| **Comment 2:** | Please change "female” or "male" to "woman” or "man" as appropriate, when used as a noun. |
| **Response:** | Thank you for pointing this out. In this revised version we changed all the times we have used "female” or "male" as a noun to "woman” or "man". As suggested, we followed the general guidelines for talking about gender with inclusivity and respect covered in Section 5.5 of the APA Publication Manual, Seventh Edition. |
|  |  |
| **Comment 3:** | We note you have included a table to which you do not refer in the text of your manuscript. Please ensure that you refer to Table 5 in your text; if accepted, production will need this reference to link the reader to the Table. |
| **Response:** | Thank you. We have ensured that all Tables and Figures included in the manuscript have a reference in the text. |

**Comments from Reviewer 1**

| **Comment 1:** | Summary: This is an ambitious paper that presents a complex analytical approach to investigate associations between a large number of individual and higher-level variables and poverty. While interesting, the paper is written in a highly technical manner at times that is somewhat difficult to understand without having a specific understanding of the statistical methods used. Further, the authors use a fair amount of statistical jargon without clearly explaining how to understand specific terms vis a vis the analysis or understand them in the context of results. |
| --- | --- |
| **Response:** | Thank you, we appreciate the comments. We have used a clearer and less technical wording in the revised paper and added explanations for the methods used. We have reworded the Materials and Methods section, particularly the part regarding the Model specification (pages 16-20 of the revised manuscript with track changes). We avoided the use of statistical jargon and specific terms. Instead, we included in this part a description of the major advantages offered by the modelling design in the context of poverty research (see lines 224-242); and, a non-technical description of the application of the boosting algorithm, stability selection and the confidence intervals (lines 288-329). |
|  |  |
| **Comment 2:** | Last, there many grammatical errors throughout that make the paper difficult to understand in places. I’ve tried to highlight a few below, but there many and the manuscript needs a very close read by a native English speaker. |
| **Response:** | Thank you. We have corrected the grammatical errors pointed out. In addition, we sent our manuscript to revision by a native English speaker professional in academic writing. |
|  |  |
| **Comment 3:** | Page 1: Grammar – “Broadly speaking, there exist [exists] a consensus on the fact that old-age...” |
| **Response:** | Change made (see line 61) |
|  |  |
| **Comment 4:** | Page 2: Grammar – “marginalization and social deprivations are associated to [with] larger [higher] poverty levels” |
| **Response:** | Change made (see line 63) |
|  |  |
| **Comment 5:** | Table 2: Several of the variables are not clearly defined. Given that much of the interpretation of the results are clearly dependent on how the variables are specified, it is important that all the variables in the model are described in detail and how any categorizations were determined. For example, please more clearly define how social networks is categorized into a single measure of three categories, how “educational lag” is defined. Similarly, “Social marginalization level in 2015.” |
| **Response:** | We totally agree. The table with the definition of variables has been restructured in the revised manuscript. As shown in pages 11-16, now we have included 5 columns: Level (individual/household, community or regional), Variable (and type of variable), Definition, Relationship types, and Source. Particularly, in the column Definition we included an explanation of the concept, a brief description of the methodology, and the definition of the categories for categorical variables. |
|  |  |
| **Comment 6:** | Results: The authors combine much of the discussion that is typically included in a discussion section into the results section. As a result, it is sometimes difficult to differentiate between what is the author’s conjecture or interpretation of the results, versus what the data suggest. The authors may want to consider including a discussion section to allow for this discussion of the results. |
| **Response:** | We fully agree and thanks you very much for this suggestion. To make this point we added a discussion section (pages 33-38) in which we have included our interpretation of the results. The results section (pages 20-33) only includes comments on the estimates, i.e. what the data suggest. |
|  |  |
| **Comment 7:** | Throughout the results section, the authors use much technical language that makes it difficult for a more general interest readership. Suggestion for the authors to define more technical terms, such as “offset” (page 17) or explain how to interpret it and why it matters. |
| **Response:** | Thank you, we agree with the comment. In the revised version, we have reworded the results section, avoiding the use of statistical / technical terms that were not contributing to the understanding or interpretation of the results. Part of the changes made is that we have included all the results in a single table, instead of having three different tables. To facilitate the lecture of Table 2 (and also following the suggestion from Reviewer 2 about highlighting the coefficients statistically different between women- and men-headed households, and among poverty levels) we only included the results with a significant effect, and we indicate in bold letters when the covariate effect on the response variable varies with household income level, and in grey __ we indicate that the covariate effects on the response variable varies according to the sex of the household head. We also included more figures to visualize the comparison between estimated effects. In this way, by focusing exclusively on the significant effects, adding shades, bold letters, and more figures, it is easier to appreciate the differences in the estimated coefficients without using technical terms.  See pages 20-33 corresponding to results, and pages 33-38 for the discussion section. Table 2, with all the results is found in pages 11-16. |
|  |  |
| **Comment 8:** | Page 12: “This association is statistically larger for poor families than for extremely poor households.” What does this mean? Did the authors do any formal statistical tests or contrasts to explore whether these coefficients are indeed statistically different? |
| **Response:** | We compare the coefficients by estimating their 95% confidence intervals. To compute the confidence intervals, 1000 random samples from the empirical distribution of the data are used. We include the confidence intervals for significant effects in Table 2, which includes a summary of the estimated coefficients for variables with stable significant effects and their 95% confidence intervals (CI). We added up to 3 decimal points to the estimations to avoid inaccuracies when comparing and referring to statistical differences. It helped us, for instance, to make more visible that the parameter estimations of the effect of covariate credit card on the response for women-headed households living in extreme poverty -0.114 [**-0.165**, -0.05] and poverty -0.224 [-0.29, **-0.163**] are not statistically different. Moreover, when commenting the results, we added the estimated confidence intervals for reference. See pages 20-33 corresponding to results. |
|  |  |
| **Comment 9:** | Page 12: “The fact that no significant correlation is found for female-headed households could be attributable to differences in socialization patterns between sexes in rural communities, which in general tend to be more traditional in terms of gender norms and roles.” The point being made here is unclear. Why would traditional gender norms and roles influence perceptions of social networks? Authors could explain this more. It would further be helpful to understand how the variable was fully defined. |
| **Response:** | In order to address your comment, we have proceeded as follows. First, as mentioned before, we added a discussion section in which we have included our interpretation of the results. Second, to make interpretations clearer we included references to previous studies in the discussion. Third, regarding the definition of variables, we have reorganized the table with the name and definition for each factor considered in the full model (see pages 11-16).  For the particular case of social networks, the definition can be found in in Table 1 (page 11 of the revised manuscript). This table also includes a reference to the official criteria for creating the three categories. Lines 527-533 include the description of the results, whereas lines 704-714 include our interpretation of the results, where we also provide references to previous studies that pointed to the existence of a different structure and composition of the social networks of women and men (lines 706 and 711). |
|  |  |
| **Comment 10:** | On page 17, the authors mention that the reader should review Table 2 for definitions of key variables. However, this is somewhat circular, as per my previous comment, several of the variables are poorly defined in Table 2. For example, “Share of female victims of violence in the workplace.” How was this variable measured? What constitutes violence in the workplace? Was this ascertained then aggregated from self report or some other means |
| **Response:** | As mentioned before, we have reformulated the table with the list of variables and included on it a definition for each one (see pages 11-16). Specifically, the variable “Share of female victims of violence in the workplace” is renamed as “Gender-based violence against women and girls in the workplace”. In the second column, the definition is “% of the 2016 women’s population aged ≥ 15 years who were victims of psychological, physical, and/or sexual gender-based violence in the workplace between October 2015 and October 2016 in the region of household residence. Expressed in decimal form.” We also included the source, the National Survey on the Dynamics of Household Relationships, and the link to the data for more details in the Bibliography. |
|  |  |
| **Comment 11:** | The paper would be improved by a clearer organization of the conclusions section. As written, it is somewhat difficult to follow, and it is unclear as to whether the importance of this paper rests primarily in the use of this specific analytical approach or if there are other important substantive results from the paper. |
| **Response:** | We fully agree and thank you for this comment, which is taken on board. We have reorganized the conclusions section as follows. First, we highlight that our paper main goal is to contribute to the studies on poverty, not the use of a specific analytical approach (lines 830-834). Second, we refer to the dataset used and the models estimated (lines 835-843). Third, we emphasized more the findings on poverty by differentiating between factors that affect equally women and men headed households and those that have a differential effect (lines 845-898). Finally, we end indicating some limitations and suggestions for further research (lines 899-905). |

**Comments from Reviewer 2**

| **Comment 1:** | This article investigates poverty using, author say, a novel approach with statistical techniques such as quantile regression and techniques to deal with high dimensionality. I think the paper should be carefully revised to make sure that readers are not lost while reading it. Also it should be made clear whether the paper is about poverty or about a technique and frame accordingly. Major revisions are expected. I think authors should make the effort as I think this work has potential and I am eager to read a second version of this paper. |
| --- | --- |
| **Response:** | Thank you for the constructive review. We agree with the comment and have made the following changes: First, we have changed the title to highlight that our paper main goal is to contribute to the studies on poverty, and not the application of the specific technique. Second, we have reworded and clarified the document, highlighting the findings on poverty. Third, we have restructured the model specification to avoid using statistical jargon. Fourth, we have restructured the results section and added a discussion section. Finally, the conclusions have been reframed to emphasize that the paper is about poverty. |
|  |  |
| **Comment 2:** | I have had difficulties to understand the dependent variable. Even though I hold a master in statistics, I think authors should not expect reader to be familiar with quantile regression as one can be with linear or binary regression models. Therefore, I think it should be carefully brought to a reader. What a quantile regression does is that for each subset of the continuous dependent variable it computes a parameter. |
| **Response:** | Thank you for pointing this out. We agree that our submission was myopic in terms of clarifying the quantile regression applied to poverty analysis. In order to make this point, we have rewritten the model specification section (lines 224-330). In lines 225-242 we clarified how the additive structure, and the quantile approach proceed. Particularly for the quantile approach, in lines 108-119 of the introduction we added a general description of the use of the quantile approach in the paper, and in lines 238-242 we provided further explanation before expressing formally the model. |
|  |  |
| **Comment 3:** | The subsets are defined by quantiles. It seems from Table 1 that there are three subsets defined at the following quantiles: 0.149 and 0.411. I do not think Table 1 is very useful, it is actually misleading as it gives the impression that the dependent variable is a categorical variable with two or three categories. I do not see the usefulness of the ‘Type of community’. |
| **Response:** | We agree, accordingly, we have deleted Table 1 (page 7) and described in the text the information on the quantiles of the dependent variable (see lines 174-194). |
|  |  |
| **Comment 4:** | Following on the previous paragraph I understand with the results that the authors have had only two quantile regressions (times 2 for gender), which I do not understand how can it be so, each X should have 3 parameters one for below the first threshold; one for below the second and above the first and one for above the second. It does not seem to be the case. Authors should explain this more clearly in the paper. This and the above paragraph can be answered together. |
| **Response:** | As explained in the last two comments, we reformulated the text about the description of the income-to-poverty variable (see lines 174-194). In this paper, we exclusively focus on two quantiles: one corresponding to people living in extreme poverty (below the extreme poverty threshold) and the other refer to people living in poverty (below the poverty threshold). |
|  |  |
| **Comment 5:** | I think the results section should be separated in three: when there are no differences in point estimate of X between the parameter for extremely poor and the one for poor. Then another part that should be emphasized because it is the main added value of the paper is that when parameters are different (when one is significant and one is not) across the two regression. Finally differences in parameter for female head and male heads are discussed/presented in a third section. I authors think differently they should explain carefully why not. |
| **Response:** | We have added the suggested structure to the manuscript. The results section was fully restructured. We reframed this, including the main table of results (Table 2 of the revised manuscript with track changes), to highlight the differences between sex of the household-head, and between poor and extremely poor households. We have proceeded as follows. First, we refer to the variables for which the estimated coefficients for women-headed and men-headed families are not statistically different (lines 376-443), and within this section we comment those whose effect is statistically different between poverty levels. Finally, in lines 446-696 we present the results in which the estimated parameters for women-headed and men-headed households are statistically different, and among them those with a statistically different parameter effect between poverty levels. |
|  |  |
| **Comment 6:** | I understand that the modelling was hard work but however hard it is, this journal is not a journal for methods in research but should show results that are relevant in public health/social science. Therefore the main contribution cannot be the hard work regarding the methods. So the paper, the whole paper, should be framed so that one or two main results regarding poverty literature should be put ahead as major contributions. The methods would obviously be in the background because it is the method that would allow this major contribution to be made. What is the key results in the poverty literature that the method allow the authors to claim as major? => I would say it should be a difference in parameter estimates between the below the threshold of extremely poor and poor therefore showing that the technique brings something. I the aim is to be have a statistical paper, then it should why this technique is new. I am not sure this is the goal. |
| **Response:** | Thank you for pointing this out. We have reframed the manuscript to emphasize that our goal is to contribute to the studies on poverty. A suggested, we highlight two major contributions. First, the identification of a subset of significant factors whose effect is independent of the head’s sex and is relevant for poor and extremely poor families. Second, the identification of a subset of significant factors with an uneven effect on income according to the sex of the head that is observed both in the poor and extremely poor households. |
|  |  |
| **Comment 7:** | Page 4: “Similarly, a person is considered to be poor if his/her income” => I would remove ‘similarly’ as it is misleading. |
| **Response:** | Change made (line 185). |
|  |  |
| **Comment 8:** | Page 4: “Based on these two lines, the dependent variable introduced” => “Based on these two thresholds, the dependent variable introduced” change lines to threshold. |
| **Response:** | Since we deleted Table 1 as suggested in Comment 3, this paragraph has also been deleted (lines 195-196). The information related is reworded in the lines 174-194. |
|  |  |
| **Comment 9:** | Page 4 “associated covariates are chosen from previous research on the matter and include a wide variety of variables at the individual, household, community, and regional levels” => “associated covariates are chosen from previous research on the matter and include characteristics at the individual, household, community, and regional levels”, shorten the variables by replacing the ‘wide variety …’ by ‘characteristics’. |
| **Response:** | Change made (line 200 and 2001). |
|  |  |
| **Comment 10:** | Page 8, ‘four quantile regression models are estimated.’ => maybe state in one small sentence which are those. |
| **Response:** | We reworded the section on the model specification. The suggestion on specifying which models are the ones estimated has been incorporated on lines 243-248. |
|  |  |
| **Comment 11:** | Page 12: “which in general tend to be more traditional in terms of gender norms and roles.” => a citation should be added for this or not try to discuss this point at this stage. |
| **Response:** | We have included a discussion section in which we link our findings to previous research. In particular, the discussion on social networks can be found on lines 704-714. The specific citation on this regard is found on line 711. We have also reworded this to clarify our point. |
