# Supplementary material for: Examining gender inequalities in factors associated with income poverty in Mexican rural households
Source: PLoS One. 2021 Nov 4;16(11):e0259187. doi: 10.1371/journal.pone.0259187 (PMC8568291; doi:10.1371/journal.pone.0259187)
Supplement: Attachment — Submitted filename: Response to Reviewers.docx [file pone.0259187.s008.docx]

Department of Economics

Georg-August-Universität Göttingen

Platz der Göttinger Sieben 3

37073 Göttingen

Germany

October 8, 2021

Dear Dr. Carlos Alberto Zúniga-González,

Thank you very much for giving us the opportunity to resubmit our revised manuscript entitled “Examining gender inequalities in factors associated with income poverty in Mexican rural households”. We are grateful to you and the reviewers for the time and effort dedicated to our document, for the improvement, precision, and quality that you brought to the manuscript.

We have been able to incorporate changes to reflect all the suggestions and comments made by you and by Dr. Simon (Jean-Baptiste) Combes. In what follows, we provide a point-by-point response to your comments and concerns, indicating the location where the changes are placed in the revised manuscript. In order to facilitate the follow-up of these changes, we have included line numbers in the revised manuscript with track changes.

We hope that the revised version fulfils your expectations and that the new structure and organization of the paper is now clearer.

Sincerely,

The authors

**Comments from the Academic Editor**

| **Comment 1:** | Please review your reference list to ensure that it is complete and correct. If you have cited papers that have been retracted, please include the rationale for doing so in the manuscript text, or remove these references and replace them with relevant current references. Any changes to the reference list should be mentioned in the rebuttal letter that accompanies your revised manuscript. If you need to cite a retracted article, indicate the article’s retracted status in the References list and also include a citation and full reference for the retraction notice. |
| --- | --- |
| **Response:** | Thank you. We reviewed the reference list. It is now correct. It was complete, however a link within the text was not working (line 105, page 4) and this have been fixed. |
|  |  |
|  |  |

**Comments from Reviewer 2**

| **Comment 1:** | Line 320 page 17: "About variable age, it has a" => "Age has a" ? |
| --- | --- |
| **Response:** | Change made. Thank you. |
| **Comment 2:** | Page 2 line 36-39: “On the other hand, most of the research is exclusively based on mean regression models analyzing the population’s average income or the expected probability of being poor, disregarding the potential inequalities across income levels. These inequalities justify the need to focus on the poorest.” => proposal : “On the other hand, most of the research is exclusively based on mean regression models analyzing the population’s average income or the expected probability of being poor, disregarding specific effects in poorer income levels. We propose to fill this gap by focusing on the poorest” <= I think this is what the paper does and the "potential inequalities" is a bit vague. I understand that the author may have try to go slowly in the introduction and to not give away too much too early, but the word "inequality" may mislead a reader as "inequality" is nearly quite a domain, I do not think the paper is actually in this domain it is in the literature on poverty (which is linked to inequality, do not get me wrong). If the paper is on inequalities I would expect that the paper looks at rich and poor while it specifically look at poor. I can change my mind if the authors bring some literature explaining carefully the link between poverty and inequalities (I am not saying it is not linked just that it is not that straightforward). |
| **Response:** | We agree with the reviewer´s assessment regarding the use of the term inequality. Accordingly, the corresponding paragraph in the revised manuscript reads as suggested by the reviewer. Thank you for pointing this out. |
|  |  |
| **Comment 3:** | Table 2, some cells are gray with nothing in it, is that on purpose? (eg. second row first column) how can be estimated the difference between men and women if the parameter is not estimated? |
| **Response:** | Thank you for this comment. Regarding the empty cells in Table 2, we have included the following clarification “Empty cells indicate that the corresponding factor is not selected as stable for that specific model, and therefore their coefficient is set to zero” in lines 247 and 2249. In this way, particularly for the case of empty cells in gray, for example for the social networks’ coefficient, this indicates that for women-headed households this is not a relevant factor (coefficient equal to zero), but for men-headed households it is relevant and statistically different than zero. Concerning the second question, as described in pages 12 and 13, about the 3-step strategy applied to overcome the model high-dimensionality, by applying the boosting algorithm a first variable selection is made, however, this selection may include some falsely selected variables. To avoid the erroneous selection of non-relevant factors we apply stability selection. The result of the application of these two steps (boosting and stability selection) is the estimation of a parsimonious model consisting exclusively of stable factors at their most suitable form (i.e. only non-zero regression coefficients), and therefore the coefficient for non-selected factors is set to zero. Setting the coefficients to zero is key since it enables the variable selection and model choice processes (to clarify this in the document, we have included this explanation in the revised manuscript, see lines 201-205 in page 12). It is important to remark that at this point of the strategy there is not an estimation of the corresponding standard errors for the stable selected factors, because confidence bands cannot be derived in the boosting estimation context. This is why we additionally conducted the pointwise bootstrap confidence intervals. These intervals are calculated only for the subset of effects selected as stable in step 2. |
|  |  |
| **Comment 4:** | Line 254 on page 15: "For extremely poor families, the coefficient for households without access to credit cards and with a woman as the head is -0.114" => can you tell us what is this parameter? it increase the income to poverty ratio by 11%? Maybe tell the reader that people with credit card are less poor? is that what it means? If I am mistaken then this comment should be taken as evidence that the paragraph is not very clear. |
| **Response:** | Thank you for pointing this out. We have reformulated this paragraph (lines 278-284 of the revised version with tracked changes) to make it clearer. The revised text reads as follows: “The results indicate that having a household member that holds a credit card is consistently linked to a greater income-to-poverty ratio in rural families. For extremely poor families, not having access to credit cards reduces their income-to-poverty ratio by 0.114 units (around 2.85 times their standard deviation) in women-headed households, and by 0.13 units by 0.13 units (more than three standard deviations) for their male-headed counterparts. The magnitude of the effect significantly varies across poverty levels, but only for men-headed households (see confidence intervals in Table 2). The estimated parameter for the effect of not holding a credit card on the ratio of income-to-poverty of poor families is -0.224 (2.39 standard deviations) for women-headed households and -0.25 (2.55 standard deviations) for those headed by a man”  In addition, we included a new paragraph about the interpretation of results in the context of quantile models and an example from Table 2 (lines 217-233): “These coefficients indicate the magnitude of the effect that each factor have on the income-to-poverty ratio. It is important to keep in mind that this ratio measures how far or close is a family to live in poverty based on their income, and therefore coefficients can be seen as estimates of the size of the effect as a proportion of the poverty line, i.e. as a share of the income required to cover the cost of the household basic food basket and the non-food basket. Interpretation of the coefficients in the quantile regression model context is basically the same as in other traditional approaches. For categorical covariates, parameters indicate the difference in the estimated effect of a category on the income-to-poverty ratio with respect to the corresponding effect of the reference category. By way of example, when examining the estimated coefficients of women-headed households living in extreme poverty, results indicate that families without access to credit cards have an income-to-poverty ratio that is smaller by 0.114 units in comparison to their counterparts having credit cards. For continuous covariates with purely linear effects, the parameter indicates the change in the income-to-poverty ratio per unit change in the continuous covariate. For instance, for men-headed households living in extreme poverty, an increase of one year in the head’s age decreases the income-to-poverty ratio by 0.003 units. For continuous covariates with nonlinear effects, interpretation is best done by visualizing the corresponding figures. By comparing the estimated coefficients from each of the models, they provide a clear picture of how the effect of the covariates varies across the poverty spectrum and according to the sex of the head.” |
|  |  |
| **Comment 5:** | Complementary to my previous comment, I think the authors should translate the parameters into meaningful effects, are parameters percentages? increases in standard deviation? at the end of the result section, page 21, line 418, it seems that the effect is an increase in the ratio, is that the case ? Then even this would not be very informative, could it be that the parameter shows the effect as the number of standard deviation of the dependent variable? With OLS it would necessitate to divide the dependent by its own standard deviation and then the parameter would be for a binarry variable like sex, being a female (if male the ref) increase by Beta (eg Beta being positive) standard deviation the dependent variable. If beta is above one then it increase by more than a standard variation , if below it increases by less than one standard variation. But I may not understand well enough the quantile regression. |
| **Response:** | Thank you. The estimated effects indicate the change in the income-to-poverty ratio, calculated by dividing household income by the poverty threshold (1715.57 MXN). Along the document (lines 43-45, 111-113, 217-233) we included comments about the response variable to better explain it and to explain the interpretation of coefficients.  We agree that there are different approaches to study poverty, thus we included comments on the advantages of this approach (lines 146-149) and a comment calling for using different indicators of poverty in further research, and the analysis of other distributional parameters of the response, such as the scale and shape (lines 610-613).  Regarding the interpretation in terms of its standard deviation, we would like to comment the following. First, our response variable, income-to-poverty ratio, is not standardized. One advantage of the quantile regression is that this is has an inherent distribution-free character since its estimation is only influenced by the local behavior of the distribution of the response, in this case the income-to-poverty ratio. We decided to analyze its distribution without reshaping nor rescaling it, but in unit terms of the ratio. Therefore, after estimating the parameters, we obtained the standard deviation (we added summary statistics in S1 Table in the supporting information section) and by dividing the parameter by the corresponding standard deviation, we can infer the importance of the estimated effects. Hence, in the results section of the revised manuscript, we commented the estimations in terms of their standard deviation to inform about the size of the effects (see pages 13-22). Thank you for this suggestion. |
|  |  |
| **Comment 6:** | line 455, page 22 When citing the world bank report on education, a little bit of the explanation of why higher education reduces fertility would be good, so could you add someting like education reduces fertility because it increases contraception and age at marriage? |
| **Response:** | Thank you. We have reformulated the text to make it clearer (lines 489-495). The updated text reads as follows: “Not holding the mandatory educational level (education lag) is another aspect of multidimensional poverty associated with income for rural poor and extremely poor families. Although more detailed statistical information is required to inquire into the reasons for this result, as stated in [49], a potential explanation could be that completing compulsory education increases income because this education level is associated with a fertility rate reduction and a health status improvement through an increase in contraceptive use and a delay of the age of marriage (or age of first pregnancy), which in turn is linked to a higher productivity.” |
|  |  |
